# Take a Break to Repair: A Dip in the World of Double-Strand Break Repair Mechanisms Pointing the Gaze on Archaea

**DOI:** 10.3390/ijms222413296

**Published:** 2021-12-10

**Authors:** Mariarosaria De Falco, Mariarita De Felice

**Affiliations:** Institute of Biosciences and BioResources, Consiglio Nazionale delle Ricerche, 80131 Naples, Italy

**Keywords:** DSB, DNA repair, homologous recombination, DNA end resection, archaea

## Abstract

All organisms have evolved many DNA repair pathways to counteract the different types of DNA damages. The detection of DNA damage leads to distinct cellular responses that bring about cell cycle arrest and the induction of DNA repair mechanisms. In particular, DNA double-strand breaks (DSBs) are extremely toxic for cell survival, that is why cells use specific mechanisms of DNA repair in order to maintain genome stability. The choice among the repair pathways is mainly linked to the cell cycle phases. Indeed, if it occurs in an inappropriate cellular context, it may cause genome rearrangements, giving rise to many types of human diseases, from developmental disorders to cancer. Here, we analyze the most recent remarks about the main pathways of DSB repair with the focus on homologous recombination. A thorough knowledge in DNA repair mechanisms is pivotal for identifying the most accurate treatments in human diseases.

## 1. Introduction

DNA double-strand breaks (DSBs) are cytotoxic DNA lesions that, if not correctly repaired, trigger genomic instability and/or cell death (a complete list of abbreviations is summarized in Table 1). Cells have developed diverse DSB repair mechanisms, among which the classical non-homologous end joining (c-NHEJ) and homologous recombination (HR) are the most represented. In addition, the cells have developed two minor additional pathways: alternative end joining (a-EJ) and single-strand annealing (SSA). These mechanisms can work in different conditions, contributing to genome rearrangements and oncogenic transformation. Even though DNA DSB repair pathways are crucial for maintaining genomic stability, if located in an inappropriate cellular context, they can cause chromosome rearrangements that can induce human diseases, spanning from developmental disorders to cancer. 

Archaea often live in extreme environments where the rate of DNA lesions is accelerated and the effect on its stability results intolerable to most life. However, they can thrive in DNA-damaging conditions, partly thanks to their robust DNA repair pathways; although, surprisingly, no DNA repair pathways unique to Archaea have been described so far.

In the present review, we examine the recent insights into the main pathways of DSB repair with the focus on homologous recombination and its various stages. Moreover, we examine the most recent advances in archaeal DNA repair processes, with particular emphasis on the molecular functions of two widely conserved archaeal proteins: Nuclease of repair in Archaea, NurA, and Helicase of repair in Archaea, HerA.

## 2. Formation and Types of DNA Breaks

Although double-stranded DNA is a stable, chemically inert molecule, genomes are continuously subjected to a plethora of exogenous and endogenous insults throughout the cell cycle [1]. While some levels of mutations are acceptable, representing the material for evolution, high levels can have serious consequences for a cell. To maintain the genome integrity, a rapid and accurate detection of the damage is therefore essential for all forms of life. If unrepaired or misrepaired, DNA damage can cause genome instability and/or cell death [2,3]. The intrinsic errors generated during the oxidative reactions of the metabolism cause the formation of the Reactive Oxygen Species (ROS) that represent one of the most important sources of DNA DSBs; these molecules are extremely reactive particles capable of damaging molecules in their immediate vicinity, including DNA.

Moreover, endogenous damages can also take place during DNA replication, recombination, and chromosome segregation, causing spontaneous hydrolysis or deamination, torsional stress of the DNA helix, and the incorporation of mismatch errors. These replication stresses may cause replication fork stalling and collapse, generating DSBs [4,5].

**Figure 1:**
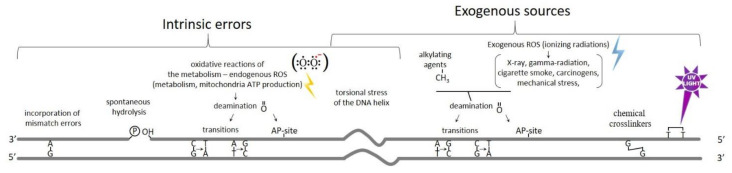
Source of DNA damage with the resulting DNA lesions. On the left are reported intrinsic errors generated during the oxidative reactions of the metabolism with the corresponding DNA damages. On the right, the exogenous sources of DNA damage, which may vary depending on the external environment, and the resulting DNA lesions.

**Figure 2:**
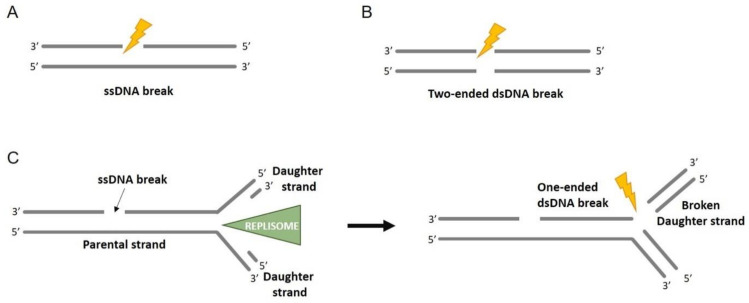
Overview of DNA breaks. (**A**) Only one strand of the dsDNA is broken; this gives rise to a ssDNA break. (**B**) dsDNA is broken into two pieces, generating a two-ended dsDNA break. (**C**) If a ssDNA break encounters a DNA replication machinery, it forms a one-ended dsDNA break.

As shown in Figure 1, the exogenous sources of DNA damage, which may vary depending on the external environment, are chemical crosslinkers, UV light, X-ray, gamma-radiation, cigarette smoke (benzo[a]pyrene), mechanical stress, carcinogens, and ionizing radiations (IR), and can induce the formation of excessive numbers of DSBs, either directly or indirectly, through the generation of ROS. The most common lesions occur only on one of the two DNA strands; this damage is known as a DNA single-strand break and, normally, it does not compromise the integrity of double-stranded DNA. However, if the lesion encounters the DNA replication machinery, the DNA single-strand break can be converted into a one-ended DNA DSB (Figure 2). Both single- and double-strand breaks can be caused by IR that can take place directly or indirectly via generation of ROS [6], causing complex lesions onto the DNA accompanied by oxidative DNA damage [7,8]. The radioactive radon gas is the most common source of accidental IR exposure; it accumulates in some locations in the basements of old homes [9]. However, IR still represents one of the most valid treatments for anticancer therapy, as it affects active dividing cells [9,10]. 

Moreover, single- and double-strand breaks can be the result of aberrant DNA topoisomerase reactions; these kinds of reactions are sometime spontaneous but can also be produced by specific inhibitors used as anticancer chemotherapeutics [11,12]. 

Notably, many widely used anticancer drugs, such as platinum salts and DNA alkylating agents, are DNA-damaging agents that suppress cancer cell proliferation by inducing various types of DNA lesions, including DSBs [13], that lead to cell cycle arrest and cell death. Therefore, the proficient repair of DSBs may grant cancer cells with resistance toward these compounds and render chemotherapy largely inefficient [14,15,16]. Increasing our knowledge on the mechanisms of DSB repair is crucial in designing more beneficial chemotherapy protocols for cancer therapy. 

Another class of DSBs are developmentally programmed breaks that occur during several cellular pathways, such as meiosis and lymphocytes recombination. Indeed, meiotic cells initiate the recombination process by carefully orchestrating hundreds of DSBs due to action of the endonuclease Spo-11, followed by rejoining mechanisms [17,18]. Thus, cells can take advantage from these events in order to generate genetic diversity. In the same way, V(D)J recombination is responsible for the immunoglobulin assembling during lymphocyte development [19].

DSBs can be either one- or two-ended DNA, depending on the mechanism of break formation. During DNA replication, the replication machinery can encounter a single strand break. This phenomenon can cause the fall apart of the replication fork and the formation of a one-ended DSB. Instead, the formation of the two-ended DSBs occurs when both the strands break at the same time or two ssDNA filaments break in the immediate proximity (Figure 2). Additionally, depending on the inducing agent, DNA ends may have different molecular structures, namely “clean” ends that, apart from the broken phosphodiester backbone, bear normal DNA chemistry, and “dirty” ends that, instead, contain additional adducts that may include anything from small chemical groups to covalently attached proteins. Only the “clean” end DSBs can be easily repaired. The DSBs caused by IR are “dirty”, while the one induced by DNA nucleases are generally “clean” [7]. Moreover, aberrant DNA topoisomerase reactions may cause a covalently attached DNA topoisomerase at the break end [20]. The formation of chromosomal aberrations is mainly dependent on the frequency and not on the end structures of the DSBs and the mechanism of DSB repair depends widely on whether the break is one- or two-ended, chemically “clean” or “dirty”, as well as the cell cycle stage.

Having in mind the intricacy of DSB formation and the resulting organism consequences, cells must have developed high specific mechanisms to deal with the genomic instability produced by DNA DSBs.

The Archaea are no exception, and indeed they often thrive within challenging environments and are thus exposed to extremes of temperature, salinity, pressure, or pH, which can increase rates of DNA damage. Some hyperthermophiles, for example, resist at temperatures that would easily denature purified DNA [21], keeping, however, similar rates of spontaneous mutation to mesophilic prokaryotes [22]. Thus, it would be expected that Archaea have especially robust DNA repair mechanisms, and this makes them a particularly interesting model system for the study of DNA repair. Surprisingly, there are many enigmas in the field of archaeal DNA repair [22,23,24] and no unique DNA repair pathways have been described.

## 3. Overview of DSB Repair Pathways

**Figure 3:**
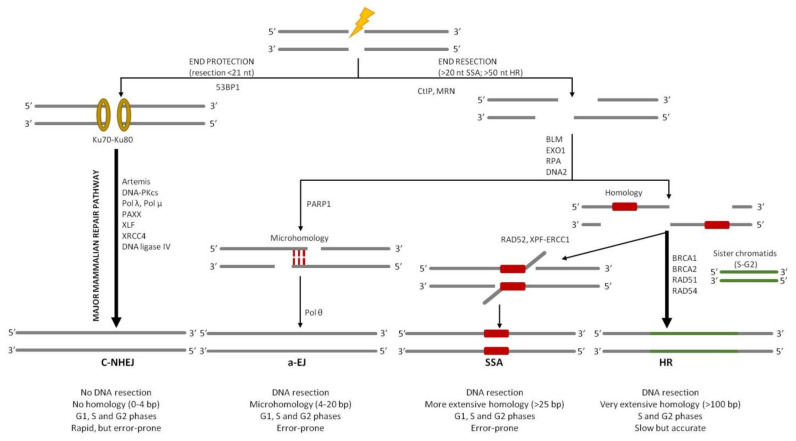
Overview of DSB repair pathways. DNA DSBs can be repaired by different pathways: the classical non-homologous end joining (c-NHEJ), alternative end joining (a-EJ), single-strand annealing (SSA), or homologous recombination (HR). c-NHEJ pathway does not require extensive end resection and microhomology regions (between 0 and 4 bp). The ends are protected from deeper resection by the binding of the Ku heterodimer (Ku70–80). a-EJ and SSA require end resection or unwinding to reveal homologous sequences, although the length of homology required is different, for a-EJ ranges from 4 to 20 bp, whereas for SSA it is longer (>25 bp). HR requires DNA end extension to generate a very extensive homology region (>100 bp). Moreover, it involves invasion of the broken DNA strands into a homologous DNA duplex molecule. This pathway is slow but very accurate, since there is no loss of genetic information.

As already said, one of the most cytotoxic and deleterious forms of DNA damage is the DSBs that, if not correctly repaired, may result in chromosome rearrangements, such as loss or deletions, translocations, and genomic instability, and negatively affect the proliferation of normal cells, causing cell death or eventually leading to the development of cancer [2,3]. Four different DSB repair pathways have been described so far (Figure 3). Although these pathways are very specific in their mechanisms and outcomes, they share many common proteins and are interdependent [5]. In most of the cases, nucleases play a crucial role in removing the damaged or mismatched nucleotides [25]. In eukaryotic cells, DSBs can be repaired mainly through two major pathways: homologous recombination (HR) [26] or classical non-homologous end joining (c-NHEJ) [27]. The choice between these two pathways is linked to the progression of the cell cycle. Since c-NHEJ does not require homology, it is active throughout all phases of the cell cycle. Instead, the need for extensive homology in HR restricts this mechanism to the S and G2 phases of the cell cycle when an identical DNA sequence, that can serve as a template for error-free DSB repair, is present in the nucleus [28]. The misregulation of these DSB repair pathways is believed to result in genome rearrangements that are typical in many cancer types; accordingly, understanding these processes is highly relevant for human health. 

HR is a high-fidelity mechanism since it relies on extensive DNA sequence homology between the broken DNA and a donor DNA molecule. This allows the recombination machinery to restore the missing genetic information in the proximity of the break site, generating error-free products; this is why it is widely conserved in all domains of life [29]. On the contrary, in the c-NHEJ pathway, the two ends of the broken chromosome are directly ligated back together with minimal reference to the DNA sequence [30]. That is why it represents an error-prone process (Figure 3). 

In addition, two alternative, less well-understood pathways for DSB repair have been defined. The first one is the alternative end joining (a-EJ), also known as microhomology-mediated end joining (MMEJ), that accommodates very limited base pairing between the two processed DNA ends, potentially forming repair joints with up to 4–20 base pairs of ‘microhomology’ [31]. The microhomology regions can bind to each other and, after removal of 3′-DNA flaps, the ends are ligated. The second alternative pathway is the single-strand annealing (SSA) that involves the annealing of more extensively processed DNA ends that carry wider homologous sequences, usually more than several tens to over a hundred nucleotides long (Figure 3). SSA represents the simplest form of homologous recombination; DSB ends are resected to produce long 3′-ended single-strand DNA (ssDNA) that can be annealed if they share a homologous sequence (Figure 3). Indeed, the human genome contains more than 500,000 short interspersed repeated sequences, so the formation of long ssDNA regions (≥5 kb in each direction) increases the probability that the exposed filament contains the repeated sequence required for the annealing. When complementary regions anneal, SSA makes a deletion between repeats as far apart as 100 kb [32,33] (Figure 3). 

Both SSA and a-EJ are mutagenic types of DSB repair since ligation of processed ends is preceded by 3′-flap excision, which generates deletions in DNA. Even though they result in being active in the S and G2 phases of the cell cycle, similarly to HR, it has been demonstrated that a-EJ can also be required in the G1 phase of the cell cycle [34]. There is still a debate if SSA and a-EJ are main DSB repair mechanisms or serve as backup mechanisms when the two major pathways, c-NHEJ and HR, are compromised in some way [5,35].

## 4. Classical Non-Homologous End Joining (c-NHEJ)

**Figure 4:**
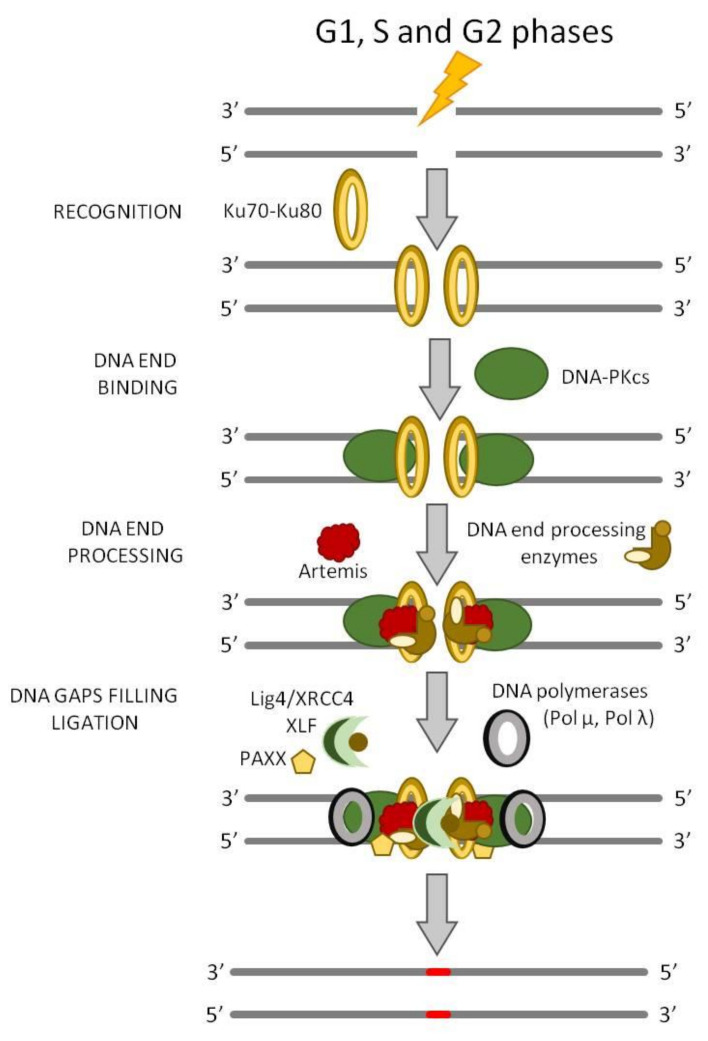
Canonical NHEJ (c-NHEJ). The c-NHEJ pathway is initiated by the Ku70–Ku80 heterodimer, it is necessary for the recruitment of the DNA-PKcs kinase. In most of the cases, the DNA broken ends are not available for a direct ligation, so they must be resected or filled before the ligation step by DNA end processing. The synthesis step required for the gap filling is catalyzed by DNA polymerase μ and λ and, lastly, the filled gap is ligated by the XRCC4–LIG4–XLF complex.

c-NHEJ is a repair mechanism that can lead to the loss of genetic information, causing short deletions at the DSB site. Moreover, c-NHEJ is template independent that means that, if multiple DSBs are present in the genome, ligation of the incorrect ends may occur, generating large deletions or chromosomal rearrangements [36]. The end-joining process can only repair two-ended DSBs, so if the break sites present abnormal structures, such as “dirty” ends (especially protein blocks), this type of repair mechanism may be inhibited. In summary, even if an end-joining pathway is a very fast DSB repair process, it is potentially mutagenic. In human cells, c-NHEJ appears to repair nearly all DSBs outside of S and G2 cell cycle phases and even about 80% of DSBs within S and G2 that are not proximal to a replication fork (Figure 4). If one or more key proteins are missing, c-NHEJ cannot take place and other DNA end-joining pathways become involved in the process. Differently from a-EJ and SSA pathways that require microhomology that ranges between 2 and 20 bp or >25 bp, respectively, c-NHEJ usually requires ≤4 bp of microhomology, although it represents the major DSB repair mechanism in most mammalian somatic cells [37,38].

Signaling factors seem to be crucial in controlling ends resection. For example, it has been demonstrated that 53BP1, the DNA damage-response protein p53-binding protein 1, acting through a number of effector proteins, is antagonistic to end resection [39,40]. Indeed, 53BP1 together with MDC1, the mediator of DNA damage checkpoint protein 1, are recruited to DSBs through modified histone residues and appear to have distinct roles in DSB repair [41,42,43]. Nevertheless, further studies have to be conducted in order to clarify how 53BP1 recruitment inhibits extensive end resection.

A key reason for the dominance of c-NHEJ is that extensive DNA end resection is prevented by Ku70–Ku80 (also known as XRCC6–XRCC5) binding that, as a matter of fact, is the first protein to take part in the process (Figure 4) [44]. Ku70–Ku80 is an abundant nuclear complex, which has high affinity for DNA ends that are either blunt or possess limited ssDNA overhangs. Long ssDNA tails have reduced affinity for Ku70–Ku80 and are directed toward c-NHEJ with less efficiency [45]. It has been demonstrated that, in vitro, several molecules of Ku can be loaded onto the DNA end, although direct imaging of Ku in living mammalian cells suggests that only one dimer of Ku normally binds to each DNA end of a chromosomal DSB [46]. A small protein called CYREN (cell cycle regulator of NHEJ, also called MRI-2, a sub-peptide of C7 orf49) has been proposed to inhibit the binding of Ku onto the DNA broken ends, thus favoring the HR pathway choice in the S/G2 phase, although the data on CYREN effects on Ku binding are conflicting [47,48].

The heterodimer Ku70–Ku80 complex binds to both the DSB ends of DNA molecules (Figure 4), acting as a platform for the further binding of DNA-dependent protein kinase catalytic subunit (DNA-PKcs), DNA ligase IV (LIG4), and the associated scaffolding factors X-ray repair cross-complementing protein 4 (XRCC4), XRCC4-like factor (XLF), and paralogue of XRCC4 and XLF (PAXX) (Figure 4) [49]. Ku70–Ku80 and DNA-PKcs establish long-range synapsis, then the two ends become closely aligned in a process requiring XLF, DNA ligase 4–XRCC4 (LIG4-XRCC4) complex, and DNA-PKcs kinase activity [50].

DNA-PKcs is a very large protein belonging to the family of phosphoinositide 3-kinase (PI3K)-related kinases (PIKKs) [50]. DNA-PKcs autophosphorylation is an important regulator of c-NHEJ, affecting Ku70–Ku80 binding but also the complex disassembly during ligation [35,50]. In addition, it phosphorylates the chromatin in the vicinity of DSBs and many downstream c-NHEJ factors, contributing to their timely recruitment and activation. The sampling of DNA end-binding partners is a dynamic process that is reversible until the process of ligation is completed. The reversible nature of c-NHEJ synapsis suggests that later steps of the pathway may also be subject to regulation. 

Blunt DNA ends or ends bearing short homologous overhangs are efficiently and rapidly sealed by the action of the LIG4–XRCC4 complex. XRCC4 is an essential binding partner of LIG4 and stimulates its enzymatic activity [51]. XLF and PAXX interact with the LIG4–XRCC4 complex and exhibit scaffolding functions, facilitating the proper positioning of DNA ends prior to ligation [52,53]. However, broken DNA ends are often not complementary and/or contain modified nucleotides, which necessitates their processing prior to ligation, often in a cyclic fashion. 

The end of the process occurs through the action of the DNA polymerases λ and μ, the nuclease Artemis, and other specialized enzymes that ensure compatibility of the ligated ends. Artemis is recruited to DSBs through its interaction with DNA-PKcs [54]. This interaction stimulates Artemis, which exhibits endonuclease activity and acts upon short overhangs or hairpins. In addition, DNA polymerase λ and DNA polymerase μ add nucleotides to the 3′-ends of the break until ligatable ends are achieved [55,56]. The repair process is not terminated until the strands of the break site are ligated, and if the DSB remains unrepaired, the repeated processing of ends may shift repair to another pathway. 

Typically, the consequence of the end process, obtained by the combined action of the aforementioned enzymes, is the drop or the gain of several nucleotides at the ends of the breaks, thus creating microdeletions or microinsertions, respectively [35]. That is why c-NHEJ is considered a mutagenic pathway for DSB repair.

### NHEJ in Archaea

Only a small number of species of Archaea possess Ku proteins and this is why it is believed that this pathway is very rare in Archaea. Indeed, only *Methanocella paludicola* is endowed with a complete NHEJ complex including Ku, polymerase, phosphoesterase, and ligase [57]. The crystal structure of these enzymes has been solved and the results obtained indicate that they are largely conserved with the bacterial counterparts [58].

## 5. Alternative End Joining (a-EJ)

**Figure 5:**
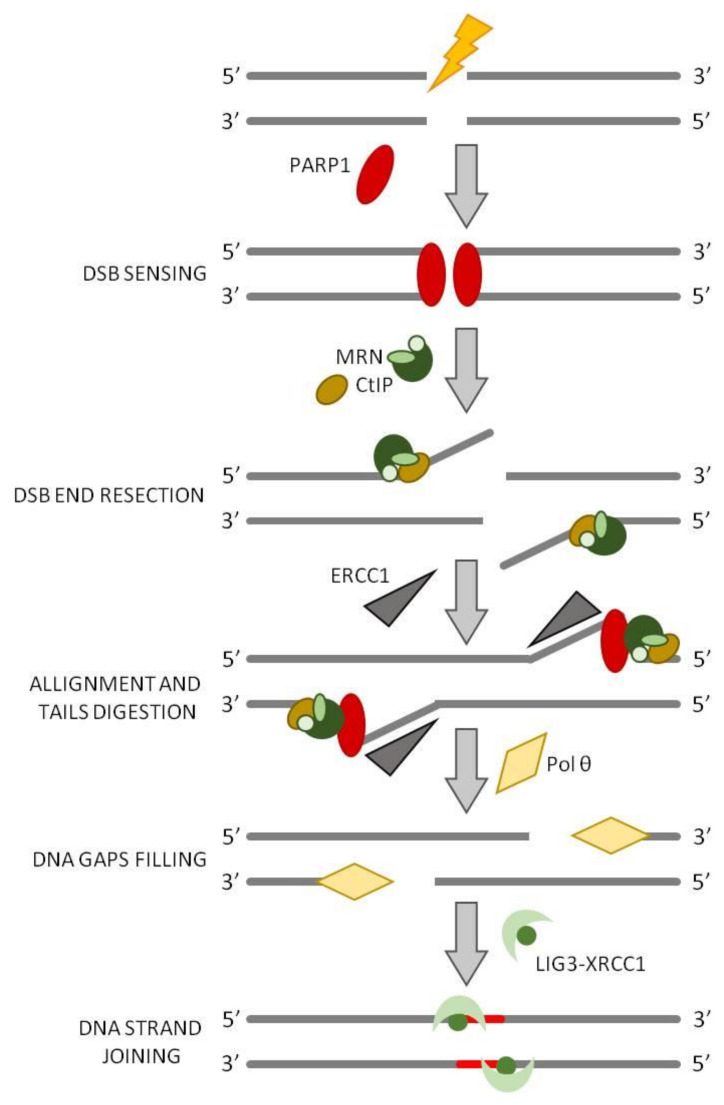
The alternative NHEJ (a-NHEJ). In a-NHEJ, the broken ends are detected and bound by PARP1, the latter recruits MRN complex and CtIP that will start the DSB end processing. DNA ends are aligned via short microhomology regions, and the resulting non-homologous 3′-tails are digested by ERCC1 nuclease. The resulting gaps are filled by Pol θ and joined by DNA Ligase3/XRCC1 complex.

It is not clear if a-EJ is a standing pathway or if the proteins working in the a-EJ are involved in other functions of dsDNA processing, such as replication, recombination, or repair, and undertake the a-EJ pathway only when c-NHEJ is compromised. Unlike c-NHEJ, a-EJ requires a 5′-DNA end resection located between 15 and 100 nucleotides from the site of the broken ends in order to expose short microhomology sequences (between 4 bp and 20 bp) that can anneal and can be used as the basis for further rejoining of the break (Figure 5) [31]. Importantly, a-EJ requires Pol θ [59,60] and may also include poly(ADP-ribose) polymerase 1 (PARP1), and the MRE11-RAD50-Nbs1, MRN, complex [61,62] and CtIP, as in HR initiation [63]. The a-EJ process starts with the endonuclease function of MRN, stimulated by phosphorylated CtIP, that generates a 3′-overhangs 15-100-nucleotide long (Figure 5). Since MRN proteins are significantly less abundant than Ku, it is supposed that MRN complex has a limited role when Ku is present [64]. It has been shown that PARP1 is involved in sensing DNA damage and promoting the a-EJ pathway [65], possibly through competition with the Ku70–Ku80 for DSB binding [65,66]. PARP1 enzymatic activity is activated by its binding to DNA and leads to the formation of long negatively-charged poly(ADP-ribose) (PAR) chains on itself and on the chromatin proteins surrounding the break (PARylation); these chains serve as a platform for the recruitment of downstream DNA repair factors [67]. Moreover, PARP1 recruits DNA polymerase θ, which is the central mediator of a-EJ [68]. Pol θ, that belongs to the A family of DNA polymerases, has a C-terminal polymerase domain and N-terminal helicase-like domain [59,69,70] that is unique among DNA polymerases. Pol θ binds to the 3′-ends of short annealed microhomology sequences stabilizing their association and extends the 3′-DNA end by using the annealing partner as a template strengthening the association between the ends of the break and creating more stable intermediate [60]. After Pol θ mediated fill-in synthesis at both sides of the annealed region, DNA ligase 1 or the LIG3–XRCC1 complex takes part of the process in order to seal the break [61]. The polymerase activity prevents further long resection, minimizing the formation of extensive deletions by SSA. Pol θ is also endowed with terminal transferase activity that is critical for the addition of nucleotides to provide microhomology that is not already present [71] https://www.ncbi.nlm.nih.gov/pmc/articles/PMC7062608/ (accessed on 16 November 2021).

### a-EJ in Archaea

This kind of pathway has been observed only in *Haloferax volcanii* [72,73] and in *Sulfolobus islandicus* [74]; however, the enzymatic basis is currently unknown.

## 6. Single-Strand Annealing (SSA)

**Figure 6:**
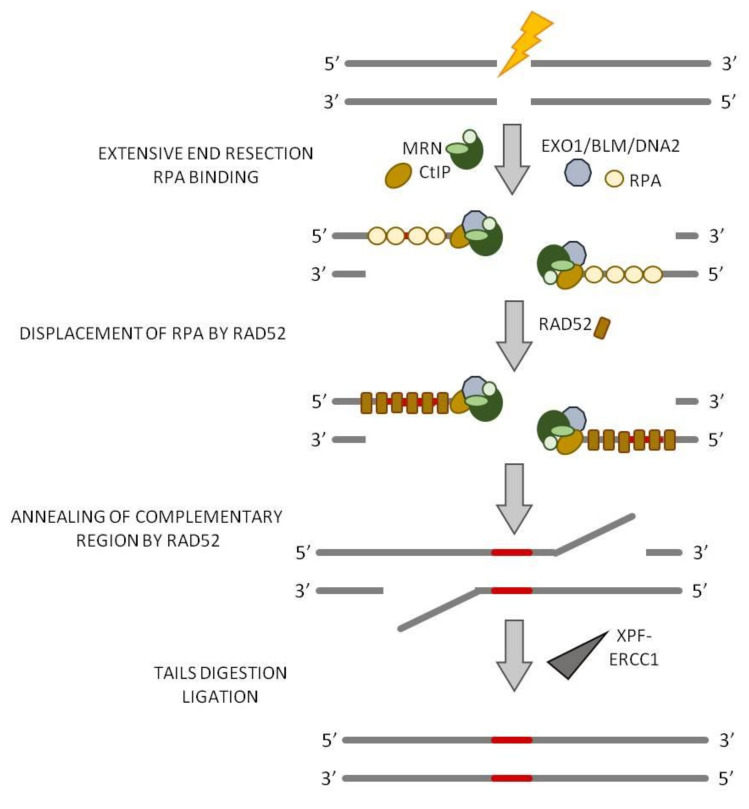
Single-strand annealing (SSA). The extensive end resection requires the action not only of MRN complex and CtIP but also of EXO1/BLM/DNA2 that are necessary to ensure a long end resection (up to 100 nt). RAD52 displaces the RPA molecules that coat the ssDNA and mediate the strand annealing between the two direct repeats. Next, the XPF–ERCC1 heterodimers remove the non-homologous flaps and, lastly, the two DSB ends are joined by DNA ligase 3.

Single-strand annealing also requires extensive 5′-end resection of DSB ends to reveal microhomology, but the extent of the resection, if compared to a-EJ, is larger since SSA may be accomplished between homologous sequences located along the 3′-ssDNA tails that are significantly longer, in the range between twenty-five and several hundred nucleotides [40]. Such homologous sequences are most often available due to the presence of tandem repeats flanking both ends of the break [5]. Neither a-EJ pathway nor SSA is reliant on Ku, and the binding of Ku to DNA ends may need to be attenuated for a-EJ and SSA to proceed. The resection necessary for the initiation of SSA is generated by MRN complex and CtIP which form 15–100-nucleotides 3′-ssDNA tails. It is at this point that the a-EJ and SSA pathways diverge. Indeed, while in a-EJ annealing of microhomology is sufficient for Pol θ to extend one of the DNA strands, to stabilize the intermediate for ligation, SSA requires the exposure of more sequence homology and, therefore, a further extension by the action of the nuclease EXO1 or Bloom syndrome RecQ-like helicase (BLM) or DNA replication helicase/nuclease 2 (DNA2) to generate longer 3′-ssDNA tails [40,75,76] (Figure 6). The long 3′-ssDNA tails are coated by multiple copies of the replication protein A (RPA) that form a filament on the ssDNA to prevent the formation of secondary structures. The annealing between the complementary ssDNA regions is mediated by the strand annealing protein RAD52, which displaces the RPA molecules coating the ssDNA (Figure 6). The unannealed region of the 3′-ssDNA tails must be processed and removed before ligation; this process requires the action of the endonucleotide excision repair complex XPF–ERCC1 (Figure 6) [77,78]. Thus, SSA is a mutagenic DSB repair pathway since the intervening sequences between the complementary regions are lost [5,35,79,80]. 

## 7. Homologous Recombination (HR)

While c-NHEJ may take place throughout the whole cell cycle, HR is largely restricted to the S and G2 phases [81]. The major conservative HR pathway in somatic cells involves recombination between sister chromatids [81,82]; sequence identity, alignment, and physical cohesion of the two sister chromatids are thought to promote sister chromatid recombination over other potential recombination partners.

Most types of homologous recombination, from Bacteria to Humans, rely on the RecA/Rad51/Dmc1 family of recombinase proteins to facilitate strand pairing and strand invasion [83,84,85]. The involvement of a larger number of enzymes makes this process more complicated, slower, but at the same time, more accurate [35,86].

HR implicates the loading of a recombinase onto ssDNA to generate extended 3’-single-stranded DNA overhangs, either at DNA ends that have undergone DNA end resection or at post-replicative ssDNA gaps, where no DSB is present [87,88]. The extended DNA end resection makes the DNA break generally non-ligatable, thus inhibiting the end-joining process and committing DSB repair to the HR-mediated pathway [89,90,91].

### 7.1. Initiation of DNA Resection

The first event is the DNA 5′-3′ end resection that plays a key role in error-free repair pathways [92]; it initially generates 3′-ssDNA, which gives rise to a platform for recruiting HR repair proteins and avoid DNA repair by NHEJ [93]. 

**Figure 7:**
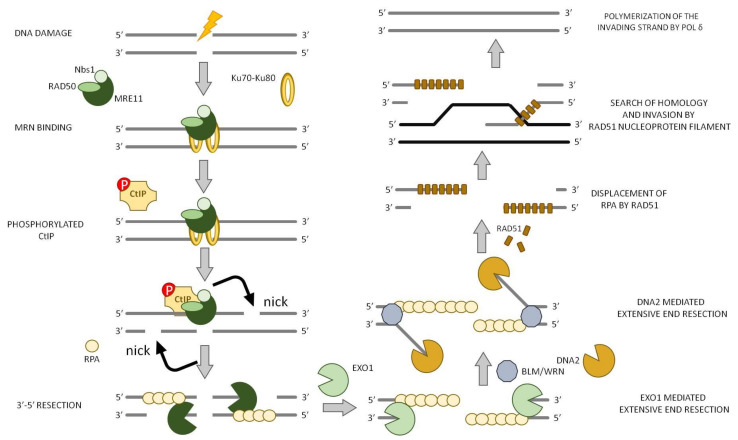
Homologous recombination (HR). MRN complex (Mre11/Rad50/Nbs1) recognizes and binds the DSB. The process starts with MRE11 that, combined with CtIP, nicks the DNA strand that possesses a free 5’-end up to 300 nt internal to the DNA, from which starts a downstream step of resection. Ku70/Ku80 block is required for an efficient initiation of “short range” resection but also to provide an entry point for “long range” resection. A 3′-ssDNA is generated in a bidirectional manner using the 3′-5′ and 5′-3′ exonuclease activities of MRE11 and EXO1, respectively, together with BLM and DNA2. Rad51 binds to the 3′-overhangs, forming a nucleoprotein filament that is responsible for the homology search and invasion step. Repair synthesis is initiated and a HJ is generated from each DNA end, which in the end of the process is resolved by the resolvase complex.

DNA end resection is a very well conserved process that depends on peculiar helicases and nucleases, some of which are specialized within a particular phylogenetic division while others are critical in all forms of life. In mammalian cells, the DSBs are initially recognized by the MRN complex that consists of three subunits: MRE11, RAD50, and Nibrin (Nbs1). Two molecules of MRE11 and RAD50 interact with each other through RAD50 walker A/B ATP-binding motif, forming a tetramer complex that binds to the lesion site (Figure 7). RAD50 possesses an extended coiled-coil tail that allows the MRN complex to form bridges with the free DNA ends. Then, ATM and CtIP are recruited to the DSB and, lastly, MRE11starts the end resection process through its nuclease activity.

MRE11 is endowed with both 3′-5′ exonuclease and endonuclease activity in vitro; its role in HR has been controversial for a long time because the polarity of MRE11 exonuclease is opposite to the direction of resection in HR and the role of MRE11 endonuclease activity was also unclear. Later on, it was demonstrated that MRE11 endonuclease activity nicks the DNA strand that possesses a free 5’-end up to 300 nt internal to the DNA. For an efficient initiation of “short-range” resection by MRE11 endonuclease activity, an interaction with CtIP (also known as RBBP8; Sae2 in *S. cerevisiae,* and Ctp1 in *Schizosaccharomyces* *pombe*) is required. Moreover, a protein block at the DNA end, such as Ku70–Ku80, RPA, or nucleosomes, further stimulates this process and also provides an entry point for “long range” resection [94,95,96,97,98,99,100,101].

The generation of the initial single-strand nick gives rise to the downstream step of resection. In this step, a ssDNA is generated in a bidirectional manner using the 3′-5′ exonuclease activity of MRE11 and the EXO1 in the 5′-3′ direction, together with BLM and DNA2 (Figure 2A) [91]. That is why both the endonuclease and exonuclease activity of MRE11 are critical for DNA end resection. 

RAD50 is a member of the “structural maintenance of chromosomes” family (SMC); it possesses ATPase activity [102] that is important for the nuclease activity of MRE11 [103]. Indeed, RAD50 strongly inhibits the 3′-5′ exonuclease activity of MRE11 in the presence of ATP [104] since the ATP binding to RAD50 induces the conformation of RAD50–MRE11 to close, thus promoting MRE11 endonuclease activity. On the contrary, the hydrolysis of ATP by RAD50 activity changes the conformation of the RAD50–MRE11 complex to an open state, favoring MRE11 exonuclease activity [105]. Thus, RAD50 acts as a molecular switch for MRE11 endonuclease/exonuclease activities.

Nbs1 modulates both the DNA binding and the nuclease activity of MRE11 [106]. Indeed, it binds phosphorylated CtIP [107] and recruits ATM by its C-terminal domain [108,109,110]. Thus, Nbs1 results in being indispensable in the mammalian system, contrary to yeast where the Xrs2/Nbs1 is not required for end resection reaction in vitro [111].

CtIP also has an essential role in DNA end resection initiation. It has been demonstrated that, in vitro, in the absence of CtIP, MRN was unable to stimulate DNA end resection [40,92].

Thus, MRE11 endonuclease associated with RAD50, Nbs1, and CtIP generates short 3′-ssDNA overhangs that are fundamental for extensive DNA end resection and RPA complex loading (Figure 7) [112,113].

### 7.2. Extension of DNA End Resection

The MRN complex, together with CtIP, generate a short (~100 nt) 3′-ssDNA overhang [114]. Since MRE11 exonuclease activity is unable to produce long 3′-ssDNA overhangs for RPA complex binding, EXO1 activity is necessary to generate extensive end resection [92]. EXO1 is endowed with 5′-to-3′ DNA exonuclease and 5′-flap endonuclease activities in vitro [115]. It is recruited to the DSB site by the MRN complex, which also stimulates its nuclease activity in vitro [111,116]. 

DNA2 is also involved in the extension of DNA end resection; it has a DNA helicase activity and a ssDNA endonuclease that cleaves only free ssDNA ends, with no nuclease effect on dsDNA. 

Another important enzyme for the extensive end resection is the RecQ helicase BLM. In fact, several studies have shown that DNA2 cleaves ssDNA via BLM-mediated DNA unwinding during end resection [117,118,119].

In addition, another RecQ family helicase (Werner syndrome ATP-dependent helicase, WRN) also functions in DNA end resection in parallel to BLM [120]. However, the different roles of these two enzymes are still not clearly understood. 

Both EXO1 and the DNA2/BLM/WRN require a short 3′-ssDNA overhang to efficiently extend DNA end resection in vitro, so they all depend on the presence of MRN complex [121].

In addition, the ssDNA binding heterotrimeric complex RPA is also required for promoting the helicase activity of BLM by coating ssDNA and regulating DNA2 nuclease activity by blocking its 3′-5′ exonuclease activity [122,123]. Thus, EXO1, DNA2, BLM, WRN, and RPA constitute the minimal complex that can carry out long-range extensive DNA end resection (Figure 7) [118]. All these proteins are evolutionary highly conserved; indeed, BLM is a RecQ DNA helicase, which is shared by Bacteria, Eukarya, and Archaea, while DNA2, which has helicase and nuclease activities, is related to the bacterial RecB proteins.

Furthermore, multiple chromatin remodeling proteins have been demonstrated to regulate the initiation or extension of DNA end resection by relaxing chromatin, thus facilitating access of the core end resection regulators to the broken DNA ends [124,125,126,127,128,129].

### 7.3. Recombination

Single-stranded DNA rapidly becomes coated with RPA in order to avoid the combination of ssDNA with other sequences, thus limiting false interactions with ssDNA intermediates of other nuclear processes [88]. Therefore, Rad51 replaces RPA from 3′-ended ssDNA overhangs and forms a nucleoprotein filament, with each recombinase subunit binding 3 nt of the ssDNA [130]. This structure has the capacity to search the homologous sequence throughout the entire genome (strand exchange) and to promote an exchange of base pairs that lead to the formation of a displacement loop (D-loop) (Figure 7) [131,132,133,134]. The Rad51 filament is a dynamic structure, subject to competing activities that promote its stability or disassembly. When Rad51 is bound to ssDNA, it undertakes a homology search that is facilitated by BRCA1-BARD1 indicating that BRCA1 promotes multiple HR steps [135]. Once the complementary region is found, it invades duplex DNA molecules and promotes the base pairing with complementary DNA sequences in the invaded molecule. Rad51 nucleoprotein filaments make synaptic complexes formed by a three-stranded DNA helix that supports heteroduplex formation-base pairing between the invading strand and the complementary strand of the invaded molecule [130]. Rad51 paralogues (Rad51C, Rad51D, XRCC2, XRCC3) co-operate with Rad51 in strand exchange reactions in vitro and are required for damage-specific Rad51 repair foci in vivo [136].

If the region of homology is sufficiently long, the synapse is stabilized and the bases that are not coupled are displaced to form a D-loop. Accordingly, the 3’-overhang of the invading strand recruits a DNA polymerase that starts the polymerization of the invading (nascent) strand using the invaded donor DNA molecule as a template (Figure 7). DNA polymerase δ (Pol δ) plays a major role in nascent strand synthesis, but translesion DNA polymerases have also been implicated in competition with Pol δ [137,138,139].

### 7.4. Termination of DNA End Resection

**Figure 8:**
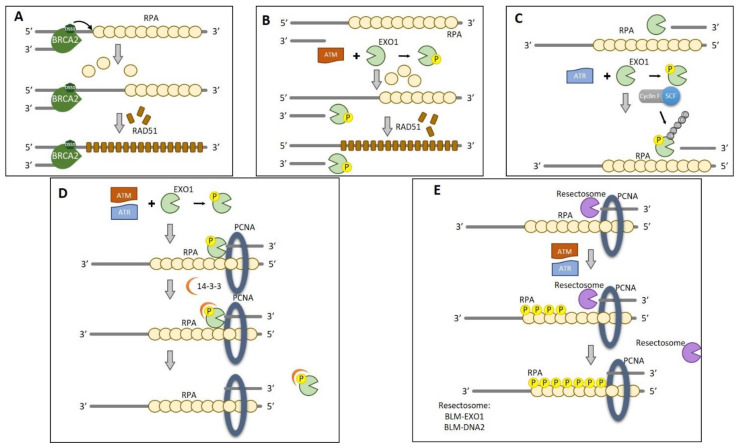
Termination of HR DNA end resection. (**A**) End-resection termination under physiological conditions. DSS1 competes with ssDNA causing the removal of RPA. Thus, Rad51 is recruited by BRCA2 and completes the switch. (**B**) Termination by phosphorylation of end resection-regulating proteins. On the contrary, EXO1 phosphorylation by ATM may regulate its activity after DNA end resection and, at the same time, can promote the dissociation of RPA from the ssDNA. (**C**) Termination by the ubiquitination of EXO1. EXO1 is rapidly degraded after the DSB induction, by the action of the Skp1-Cullin1-F-box family of ubiquitin ligases in a proteasome-dependent manner. (**D**) Termination by 14-3-3 interaction. The phosphorylation motif binding protein 14-3-3 causes the disruption of EXO1-PCNA interaction, facilitating end resection in an ATM/ATR-dependent manner. (**E**) Termination by RPA phosphorylation. The unphosphorylated RPA complex promotes an early extensive resection by BLM-EXO1 and BLM-DNA2 resectosomes. On the contrary, the phosphorylation of RPA by ATM/ATR induces BLM strand switching, giving rise to end resection termination.

Uncontrolled end resection is toxic to cells since it causes the loss of genetic information [140]. For example, unlimited end resection may reduce the efficiency of RPA recovery leading to an increase in ssDNA exposure, replication fork collapse, and genomic instability [141]. Accordingly, the end resection must be terminated as soon as the length of ssDNA is sufficient for HR repair. The regulation of DNA end resection termination is still unclear, although diverse mechanisms have been proposed. Under physiological conditions, end resection is terminated by the action of BRCA2-DSS1 that allows Rad51-RPA switch. DSS1, a small 70 residues protein, competes with the ssDNA thanks to its high acidic residues content, causing the removal of RPA from real ssDNA. Thus, Rad51 is recruited by BRCA2 and completes the switch [142] (Figure 8A).

The phosphorylation of EXO1 by ATM may regulate the activity of EXO1 after DNA end resection and, at the same time, promotes the dissociation of RPA from the ssDNA (Figure 8B) [143,144,145]. Another hypothesis indicates that termination can occur by the targeting of end resection-regulating proteins. It has been demonstrated that, in mammals, EXO1 is rapidly degraded after the DSB induction, by the action of the Skp1-Cullin1-F-box family of ubiquitin ligases in a proteasome-dependent manner (Figure 8C); other studies suggest that ATR-mediated EXO1 phosphorylation also promotes EXO1 degradation [143]. 

It has also been suggested that the disruption of the EXO1–PCNA interaction, due to the phosphorylation motif binding protein 14-3-3, may attenuate exonuclease activity, facilitating end resection in an ATM/ATR-dependent manner (Figure 8D) [146,147]. Taken together, these data suggest that ATM/ATR can be involved in the end resection termination through different pathways. In addition, in 2016, Tkáč et al. [148] reported that DNA helicase B (HELB), that translocates ssDNA in the 5′-3′ direction, is able to inhibit BLM-DNA2, and EXO1 nuclease activity [148]. Nevertheless, the mechanism by which HELB functions is still not clear.

Recently, Ilya Finkelstein’s group demonstrated, through in vitro experiments, that the unphosphorylated RPA complex stimulates the initiation of the BLM-EXO1 and BLM-DNA2 resectosomes, promoting an early extensive resection (Figure 8E) [149]. On the contrary, the phosphorylation of RPA by ATM/ATR gives rise to end resection termination; indeed, it induces BLM strand switching when the nuclease is omitted from the reaction. However, these observations need to be further valuated in vivo. 

Moreover, recent studies also suggest that RNAs may have a role in end resection termination [150,151].

However, it is still unclear if this mechanism takes place in all cells or in specific sites, such as in transcriptively active ones. 

Furthermore, it has been suggested that some proteins involved in c-NHEJ can also be involved in the end resection termination. For example, the SHLD2, one of the four subunits of the shieldin complex, can bind ssDNA instead of RPA, thus blocking end resection inhibiting nuclease/helicase recruitment [5,152].

## 8. Homologous Recombination in Archaea

The best studied DSB repair process in Archaea is the HR [153]. It is involved not only in DNA repair [154,155,156] but is also used to promote the genetic diversity thanks to the DNA transfer between *Sulfolobus* [157,158,159] and *Haloferax* species [160,161]. Moreover, HR is also involved in the restart at the stalled forks that may occur during DNA replication caused by a DNA damage or a protein roadblock. 

Even in Archaea, several homologous of eukaryotic HR proteins have been identified, including MRE11, RAD50, and the recombinase RadA [162]. It has been demonstrated that, in *Sulfolobus acidocaldarius*, the Mre11–Rad50 complex undergoes post-translational methylation in response to γ-irradiation [163]; it has also been seen that, in *H. volcanii,* Mre11–Rad50 complex acts in both the repair of DSBs and the compaction of the nucleoid after DNA damage [72,164].

**Figure 9:**
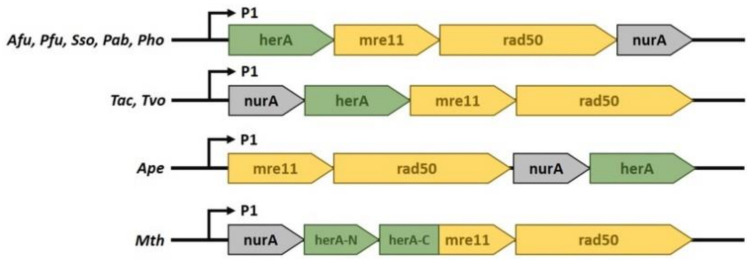
Genomic localization of HerA, NurA, Mre11, and Rad50 genes from representative species in Archaea. *Archaeglobus fulgidus* (*Afu*), *Pyrococcus furiousus* (*Pfu*), *Sulfolobus solfataricus* (*Sso*), *Pyrococcus abyssii* (*Pab*), *Pyrococcus horikoshii* (*Pho*), *Thermoplasma acidophilum* (*Tac*), *Thermoplasma volcanium* (*Tvo*), *Aeropyrum pernix* (*Ape*), *Methanobacter thermoautotrophicus* (*Mth*).

Moreover, two widely conserved archaeal genes, *herA* and *nurA*, have been implicated in HR since they are usually encoded in the same operon of *Mre11* and *Rad50* (Figure 9) [165,166,167] and the four genes are co-induced in response to UV irradiation [154,168]. Several studies have demonstrated that DNA resection requires the cooperation between the ATP-dependent Mre11-Rad50 and HerA–NurA complexes [169,170,171,172]. HerA and NurA are believed to be the functional homologs of the eukaryotic Dna2/DNA2, Exo1/EXO1, and Sgs1/BLM proteins [173]. Bacterial homologues of NurA and HerA have been identified in *Deinococcus radiodurans*, as well as in Archaea [166,174,175,176].

### 8.1. End Resection

**Figure 10:**
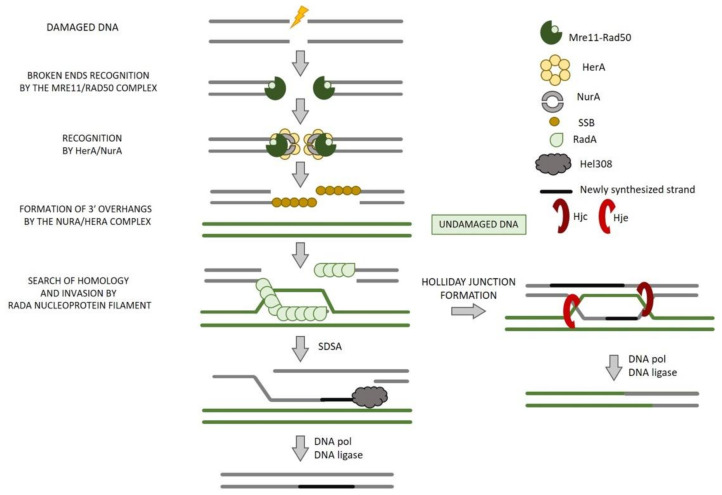
Homologous recombination-based DSB repair in Archaea. Recognition of DNA damage by Mre11/Rad50 complex allows the formation of 3′-overhangs by the NurA/HerA complex. The ssDNA is coated by the SSB protein that is then replaced by RadA. RadA forms a nucleoprotein filament on the 3′-overhangs that is necessary for the identification of the homologous region and for the strand invasion process. If only one strand invasion event occurs before repair conclusion, the resulting non-crossover event is known as synthesis-dependent strand annealing (SDSA). On the contrary, if both strands are involved in strand invasion, we observe the formation of a Holliday junction, the resolution of which may lead to crossover events.

The sensing and the processing of DSB ends occurs by Mre11–Rad50 complex that, through their 3′-5′ exonuclease and endonuclease activities, makes short 3′-overhangs (Figure 10) [170,177]. The combined action of HerA and NurA produces long 3′-overhangs. The connection between Mre11/Rad50 and NurA/HerA, necessary to produce appropriate resectioning, remains not clear and is fundamental for understanding the initiation of DSB repair by HR in Archaea.

### 8.2. NurA and HerA

The molecular functions of archaeal NurA and HerA have been analyzed in detail. NurA has Mn^2+^-dependent 5′-3′ exonuclease and also has an endonuclease activity on single- and double-stranded DNA [165,166,176,178]. HerA is a bipolar (5′-3′ and 3′-5′) ATP-dependent helicase that is promoted by the binding to ssDNA and dsDNA [179].

Crystallographic studies indicate that the high-resolution structure of HerA from thermophilic archaeon *Saccharolobus solfataricus* co-crystallize with a non-hydrolysable ATP analog (AMP-PNP). Six monomers of HerA are assembled as a hexameric ring, stabilized through the interaction of a conserved C-terminal brace between adjacent HerA protomers [171].

NurA forms homodimer arranged in a toroidal configuration of RNaseH-like domains by intertwining the helical N- and C-terminal extensions. NurA dimer has a central channel lined with opposed active sites large enough to accommodate up to two strands of unwound DNA. In addition, the positively charged residues present in the inner surface of the channel facilitate the interactions with the phosphodiester chains of DNA [175]. It has been observed that HerA bounds around the strands of the dsDNA, whereas the dimeric nuclease NurA is thought to preferentially bind onto the outside of the hexameric HerA-dsDNA substrate which is mediated by the N-terminal HAS (HerA and ATP synthase) domain [171]. In complex with the HerA hexamer, the NurA dimer generates a continuous channel, indicating that HerA-driven translocation pushes the DNA duplex through the channel of NurA, where it is unwound and degraded [171,172]. HerA ring stimulates 5′-3′ exonuclease activity of NurA [166,172,175,176,180], likely by coupling translocation and ssDNA substrate presentation for NurA to degrade. Furthermore, activities of NurA and/or HerA are stimulated by the Mre11 or Mre11–Rad50 complex [170,181]. It was also found that HerA is essential for cell viability in *S. islandicus* [156]. It has been also demonstrated that nucleotide-free HerA in solution is in equilibrium between hexameric and heptameric forms, with a large dominancy of the heptamers. The heptameric form can only bind dsDNA in the absence of nucleotides, whereas the hexameric form can bind dsDNA independently from the nucleotide [172].

NurA and HerA homologs are also conserved in many kinds of Bacteria, such as Cyanobacteria, Actinobacteria, Firmicutes, and *Deinococcus Thermus*, but they are less well understood than the archaeal ones; indeed, they have been studied only in *D. radiodurans.* In the latter, NurA shows 5′-3′ ssDNA/dsDNA exonuclease/endonuclease activities only in the presence of HerA and the ATPase activity of HerA is also stimulated by interaction with NurA [166] and, in addition, HerA interacts with RecJ, stimulating its 5′-3′ exonuclease activity [174]. Differently from archaeal *nurA* and *herA* genes, the *D. radiodurans* counterparts do not form a gene cluster with the homologous genes of *Mre11* and *Rad50*. Interestingly, in some Archaea, NurA and HerA may function as CRISPR-associated proteins [182]. The CRISPR viral defense systems have been found in ~85% of Archaea. Cas enzymes generate guided DSBs that are eventually repaired by non-HR pathways, NHEJ, and MMEJ [183]. Studies on non-HR DSB repair has permitted the development of the first type II CRISPR-Cas-based genomic editing systems in Archaea [74,184].

### 8.3. Strand Invasion and Exchange

Immediately after end resection accomplishment, a lot of proteins take part in the homology search, strand invasion, and exchange steps that give rise to the formation of the Holliday junctions (HJ). 

The first protein is the single-strand binding protein SSB (corresponding to the eukaryotic RPA) that acts as a DNA damage sensor but also as ssDNA chaperones during many pathways such as DNA replication, repair, and recombination. 

In Euryarchaea, SSB is heterotrimeric resembling the eukaryal RPA [185], whereas in Crenarchaea, it is monomeric in solution [186]. During HR, SSB binds to the 3′-ssDNA ends generated by the complex composed by Mre11-Rad50-NurA-HerA and is then replaced by RadA that forms a nucleoprotein filament necessary for the strand exchange. If the invasion involves only one strand, synthesis-dependent strand annealing (SDSA) can occur before repair conclusion, resulting in a non-crossover event. On the contrary, a HJ may be formed after the invasion of both strands, the resolution of which may lead to crossover events (Figure 10). 

RadA is a DNA recombinase and belongs to the RecA protein family (such as RAD51 in Eukarya and RecA in Bacteria). The archaeal RAD51 protein is similar to the eukaryal counterpart RAD51, sharing the dsDNA-binding N-terminal domain and lacking the RecA-specific C-terminal domain [187]. The deletion of RadA in *H. volcanii* leads to severe DNA recombination, repair, and growth defects [72]. Most archaeal genomes encode one or more RadA paralogues; the first to be characterized was RadB that does not support strand exchange in vitro [188,189]. In addition, genetic studies indicate that it has an important role in recombination and repair [189]. *S. solfataricus* possesses three RadA paralogues, all expressed at a lower level than RadA. One of these paralogues, *Sso*2452, has been suggested to have an anti-recombinase activity; therefore, it may have a critical role in limiting HR in vivo. Indeed, it has been demonstrated that it is able to bind ssDNA more tightly than RadA, inhibiting D-loop formation in vitro, even when the RadA nucleoprotein filament is pre-formed [190].

### 8.4. Branch Migration

A molecular motor responsible to provide directionality to strand exchange has only been identified in Bacteria and is known as RuvAB; no equivalents have been identified in Eukarya. In Archaea, the best candidate for this role is the helicase Hel308, also known as Holliday junction migration, Hjm, that is also widely conserved in Eukarya. Archaeal Hel308 has a faint HJ branch migration activity in vitro but can unwind branched DNA structures [191,192]. The role of Hel308 in HR was controversial since its properties suggest a role in replication fork restart more than in branch migration; however, the hypothesis of its involvement in HR was reinforced by the indication of its physical and functional interaction with the HJ-resolving enzyme, Hjc (HJ cleavage) [193].

### 8.5. HJ Resolution

It has been demonstrated that Archaeal Hjc have properties that are analogue to the *E. coli* resolving enzyme RuvC, even if they do not share any sequence similarity [194]. In addition, in 2000, Kvaratskhelia et al. have identified a homologue of Hjc, named Hje (HJ endonuclease) [195]. The two enzymes do not possess a detectable sequence preference. Moreover, they cleave the HJs at a different position with respect to the junction center and on a different pair of arms, suggesting that Hjc and Hje differ in the way they manipulate the junction on binding [196] (Figure 10).

## 9. Perspective on DSB Repair in Archaea

Together with Bacteria and Eukarya, Archaea represents one of the three domains of life [197]. There has long been growing interest on DNA repair mechanisms in Archaea, and the explanation for this may lie in two main reasons. The first one is related to the fact that many Archaea inhabit extreme environments, and they have to afford the accelerated rate of physical damage to DNA. This suggests that Archaea might reasonably possess particularly robust or novel DNA repair pathways to deal with this phenomenon. Secondly, in spite of the morphological difference existing between Eukarya and Archaea, the latter share certain fundamental molecular features with eukaryotic cells. In particular, it is now generally accepted that the Archaea share many similarities in their information-processing systems with Eukarya; indeed, Archaeal and eukaryal DNA replication, repair, and transcriptional machineries show particularly striking similarities [198]. For this reason, most of the early studies on DNA repair enzymes in Archaea arose from the researchers’ desire to analyze the DNA repair process in simpler model systems in order to gain useful information on their eukaryotic counterparts (ultimately, human). Archaeal models have helped to build a picture of how DNA repair mechanisms operate to maintain genomic stability. For instance, they provided significant information on the workings of the highly conserved Mre11–Rad50 complex [199,200,201,202,203]. Further insights will arise from the forthcoming investigations on DNA end-resection pathways in Archaea and other model systems. DNA damage repair research will offer an extraordinary chance to achieve a deeper knowledge on the integrity of genomic DNA, thus promoting clinical disease prevention and therapeutic options, especially boosting the precise cancer therapy [204,205]. The growing information on DNA repair mechanisms opened up a new track therapy on precisely targeted cancer. This is why we believe that precise and in-depth research into the molecular mechanisms of DNA damage, response, and repair, using model organisms such as Archaea, will drive significant advances in the near future and, hopefully, will lead to the achieving of more robust clinical trial results.

In addition, since many Archaea live in environmental extremes, they may have developed mechanisms not represented in bacteria or eukaryotes, thus representing a potential source of new information concerning early cellular evolution, as well as processes that may be used for biotechnology. In fact, new enzymes will continue to be identified and studied in order to find new roles in the biotechnology world. One of the most striking examples is the developing analysis on the relation between DSBR and CRISPR systems that will surely revolutionize medicine but also agriculture [206,207]. Indeed, it has the potential to cure a range of genetic diseases and, at the same time, to edit plant genome for the production of more healthy and productive plants as well as for the prolongation of the shelf-life of many perishable foods.

## 10. Conclusions

Here, we have reviewed the current knowledge regarding the different pathways used for the repair of DSBs, emphasizing the homologous recombination process in Eukarya and Archaea. DSBs represent the most cytotoxic DNA lesions that, if not correctly repaired, cause genomic instability and/or cell death. Cells possess multiple repair mechanisms, but HR is the only high-fidelity repair process. The complexity of HR machinery increases with that of the organism. This is why studies in simpler systems, such as Archaea, may be a valid approach to establish paradigms that can help to understand the more complex human HR pathway. Ongoing research into HR will be crucial to paint the full picture of this elegant DNA repair mechanism and, accordingly, will provide additional insights into the origins of cancer that will help to identify new therapeutic targets in diseases that are characterized by genomic instability.

## Figures and Tables

**Table 1:** List of abbreviations used in this article.

Abbr.	Definitions	Abbr.	Definitions
53BP1	DNA damage-response protein p53-binding protein 1	MDC1	mediator of DNA damage checkpoint protein 1
a-EJ	alternative end joining	MMEJ	microhomology-mediated end-joining
ATM	ataxia telangiectasia mutated	MRN	Mre11-Rad50-Nbs1
ATR	ataxia telangiectasia and Rad3-related protein	Nbs1	Nibrin
BLM	Bloom syndrome RecQ-like helicase	NurA	Nuclease of repair in Archaea
BRCA1, 2	Breast cancer 1, 2	PAR	poly(ADP-ribose)
Cas	CRISPR-associated	PARP1	poly(ADP-ribose) polymerase 1
c-NHEJ	classical non-homologous end-joining	PAXX	paralogue of XRCC4 and XLF
CRISPR	clustered regularly interspaced short palindromic repeats	PCNA	Proliferating cell nuclear antigen
CtIP	C-terminal binding interacting protein	PI3K	phosphoinositide 3-kinase
DNA2	DNA replication helicase/nuclease 2	PIKKs	PI3K-related kinases
DSB	DNA double-strand breaks	PKcs	DNA-dependent protein kinase catalytic subunit
DSS1	deleted in split hand/split foot	Pol δ	DNA polymerase δ
ExoI	Exonuclease I	RAD51	Radiation sensitive 51
HELB	DNA helicase B	ROS	Reactive Oxygen Species
HerA	Helicase of repair in Archaea	RPA	replication protein A
HJ	Holliday junction	Ruv	Recombination UV
Hjc	Holliday junction cleavage	Sgs1	Slow growth suppressor 1
Hje	Holliday junction endonuclease	SHLD2	Shieldin 2
Hjm	Holliday junction migration	SMC	structural maintenance of chromosomes
HR	homologous recombination	SSA	single-strand annealing
IR	ionizing radiations	SSB	Single-strand binding protein
LIG4	DNA ligase IV	WRN	Werner syndrome ATP-depenent helicase

## Data Availability

Not applicable.
